# Flexible Sensory Platform Based on Oxide-based Neuromorphic Transistors

**DOI:** 10.1038/srep18082

**Published:** 2015-12-11

**Authors:** Ning Liu, Li Qiang Zhu, Ping Feng, Chang Jin Wan, Yang Hui Liu, Yi Shi, Qing Wan

**Affiliations:** 1 School of Electronic Science & Engineering, Nanjing University, Nanjing 210093, People’s Republic of China; 2 Ningbo Institute of Materials Technology and Engineering, Chinese Academy of Sciences, Ningbo 315201, People’s Republic of China

## Abstract

Inspired by the dendritic integration and spiking operation of a biological neuron, flexible oxide-based neuromorphic transistors with multiple input gates are fabricated on flexible plastic substrates for pH sensor applications. When such device is operated in a quasi-static dual-gate synergic sensing mode, it shows a high pH sensitivity of ~105 mV/pH. Our results also demonstrate that single-spike dynamic mode can remarkably improve pH sensitivity and reduce response/recover time and power consumption. Moreover, we find that an appropriate negative bias applied on the sensing gate electrode can further enhance the pH sensitivity and reduce the power consumption. Our flexible neuromorphic transistors provide a new-concept sensory platform for biochemical detection with high sensitivity, rapid response and ultralow power consumption.

With the recent interest in brain/computer interfaces^1^, soft robotics^2^, wearable electronics^3^ and skin-like sensory systems^4^, flexible devices have attracted growing attention. These emerging devices require new fabrication schemes that enable integration with soft, curvilinear and time-dynamic human tissues. Among these devices, flexible sensors are becoming increasingly significant in a wide-variety of novel applications such as *in vivo* monitoring^5^, delivery of advanced therapies^6^, artificial sense organs^7^, etc. As a fundamental component for sensor application, field-effect transistors (FETs) based sensors have been intensively investigated due to their inherent advantages of miniaturization, facilitated integration, direct transduction and label-free detection^8^,^9^,^10^,^11^. The classical sensing mechanism of the FET-based sensor is attributed to a charge-dependent interfacial potential due to the adsorption of potential-determining species at sensing membrane/electrolyte interface^12^. The sensitivity is limited to ~59.2 mV/decade (Nernst limit) at room temperature when the threshold voltage (V_th_) is recorded as the output signal. It should be noted here that above mentioned measurements are based on the quasi-static electrostatic coupling mode, which potentially increases the time consumption and energy dissipation. But in smart sensory platforms, such as implantable devices and wearable sensory systems, low power consumption is one of the most important pre-requisites.

Synergic integration of presynaptic inputs from the dendrites plays an important role for sensory information process and cognitive computation, and the idea of building bio-inspired solid-state devices has been around for decades^13^,^14^. In 1992, Shibata *et al.* proposed Si-based neuron transistors with multiple input gates that are capacitively coupled to a floating gate^15^. The “on” or “off” state of the neuron transistors depends on the integrated effect of the multiple input gates. One of the unique features of the neuron transistors is the ultralow power dissipation during calculation due to the gate-level sum operation in a voltage mode. From then on, Si-based neuron transistors have attracted much attention for chemical and biological detection due to the easy adjustment of threshold voltage^16^,^17^,^18^,^19^,^20^. When an asymmetric gate capacitor structure is adopted, magnification of V_th_ shift can be observed in the neuron transistor when the sensing gate experiences a load from electrolyte. This device concept scales up the surface potential shift by the capacitance ratio between the sensing gate and the control gate^21^,^22^,^23^. But, up to now, flexible electrolyte-gated neuron transistors with amorphous oxide channel layers for biochemical sensing applications have not been reported.

Amorphous oxide-based transistors were proposed as promising fundamental unit in sensory platform due to their low process temperature, superior electrical properties, high reliability and easy reproducibility^24^,^25^,^26^. To date, remarkable sensing performances have been demonstrated in these oxide-based transistors^27^,^28^,^29^. For portable applications, low-voltage operation is preferred. Electrolyte gated electric-double-layer (EDL) transistors can act as potential candidates with a low operation voltage due to the strong EDL modulation at the electrolyte/channel interface^30^,^31^. Recently, oxide-based EDL transistors gated by solid-state inorganic electrolytes were proposed by our group^32^,^33^. At the same time, artificial synapses and neuromorphic transistors with low power consumption and fundamental biological functions were mimicked in these devices^34^,^35^,^36^. In the present work, flexible sensory platform based on individual protonic/electronic coupled indium-zinc-oxide (IZO) neuromorphic transistor was fabricated on plastic substrates. Such neuromorphic transistor exhibited a high sensitivity when a quasi-static dual-gate synergic modulation mode was adopted. Most importantly, single-spike dynamic sensing of such flexible neuromorphic transistor was also investigated, and pH sensing with ultra-high sensitivity, very quick response/recover time, and extremely low power consumption were realized.

## Results

**Figure 1:**
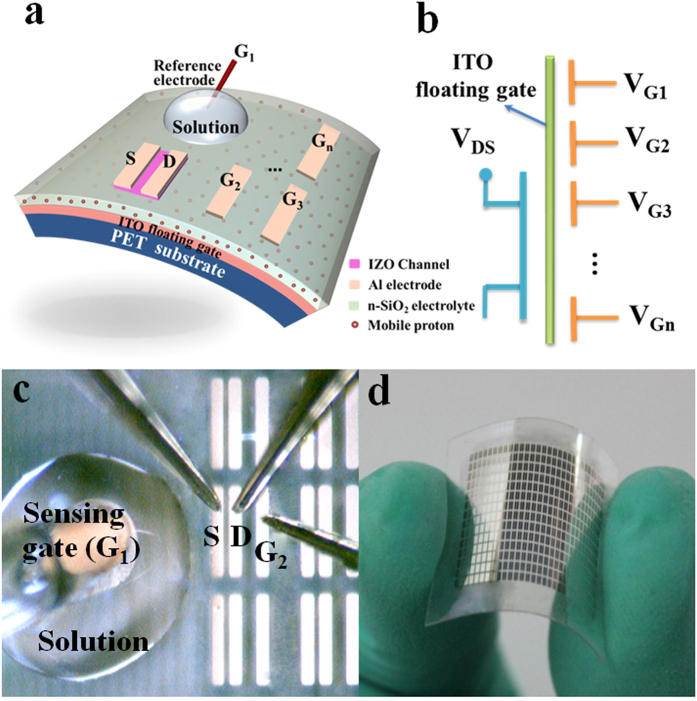
IZO-based neuromorphic transistor on PET substrate and its flexibility exhibition. (**a**) Schematic of the flexible pH sensor based on an IZO neuromorphic transistor with multiple gate electrodes. An Ag/AgCl reference electrode immersed in the solution droplet acts as the sensing gate. In-plane Al electrodes are used as the control gates. (**b**) The schematic image of the capacitive network of the flexible neuromorphic transistor. The carrier density of the IZO channel is modulated by the weighted sum of all inputs of sensing gate and control gates. (**c**) An optical microscope image of the system (Taken by Dr. Ning Liu). (**d**) The sensor array fabricated on a flexible PET substrate (Taken by Dr. Ning Liu).

Figure 1a shows the schematic diagram of a flexible IZO-based neuromorphic transistor with multiple in-plane gate electrodes for pH sensing application. A miniature Ag/AgCl reference electrode immersed into a 5.0 μL pH buffer solution droplet on the nanogranular SiO_2_ (n-SiO_2_) electrolyte film acts as a sensing gate (G_1_). In-plane aluminum (Al) electrodes (G_2_, G_3_…G_n_) are used as control gates. The distinctive feature of our device is that sensing gate and all control gates are located at the same plane. The capacitive network of the neuromorphic transistor is plotted in Fig. 1b. The carrier density of the IZO channel layer can be electrostatic modulated by the weighted sum of all inputs from the sensing and control gates. The weight for each gate is directly proportional to the capacitive factor normalized by the total capacitance of the floating gate^5^. Figure 1c displays the top-view optical image of the IZO-based neuromorphic transistor sensor. The channel width (W) and length (L) is 1000 and 80 μm, respectively. As a proof of concept, only one sensing gate and one control gate electrode is used in the present work. The distance between the control gate electrode and the drain electrode is 300 μm. Figure 1d shows a picture of the IZO-based neuromorphic transistor array on PET plastic substrate, exhibiting its flexible nature under external force.

**Figure 2:**
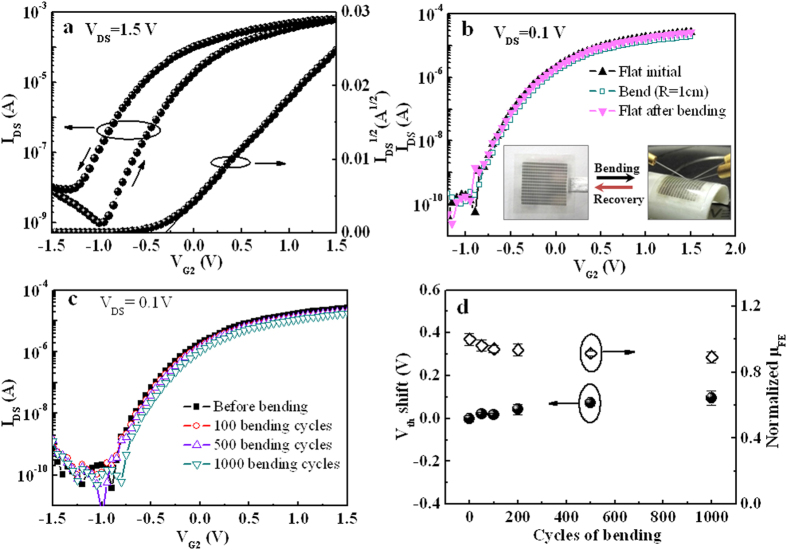
Electrical properties the IZO-based neuromorphic transistor and its flexibility characteristics. (**a**) Transfer curves of the IZO-based neuromorphic transistor measured by sweeping the voltage on the control gate (G_2_) at V_DS_ = 1.5 V. An anticlockwise hysteresis loop of ~0.4 V is observed. (**b**) Transfer curves of the flexible neuromorphic transistor measured before, during and after bending by a cylinder with a radius of 1.0 cm. The inset is the pictures during the measurement process (Taken by Mr.Ning Liu). (**c**) Transfer curves of device measured before and after repeated bending cycles by sweeping the control gate (G_2_) at V_DS_ = 0.1 V. (**d**) The variations in V_th_ and μ_FE_ of the flexible neuromorphic transistor with repetitive bending cycles. Error bars represent standard deviations for 5 samples.

Figure 2a shows the transfer characteristics of the IZO-based EDL transistor at a constant V_DS_ of 1.5 V. Gate voltage applied on lateral Al gate electrode is swept from −1.5 V to 1.5 V and then back. A clear anticlockwise hysteresis window of ~0.4 V is observed, which is likely due to the mobile protons within the nanogranular SiO_2_ electrolyte^37^. Subthreshold swings (SS), current on/off ratio (I_on_/I_off_) and V_th_ are estimated to be ~175 mV/decade, ~6.4 × 10^5^, and −0.3 V, respectively. In addition, field-effect electron mobility (μ_FE_) at the saturation region is estimated to be ~12 cm^2^/V.s by the following equation:


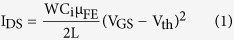


where C_i_ (~2.7 μF/cm^2^) is the specific capacitance of the SiO_2_ electrolyte measured from two in-plane Al gate electrodes at 1.0 Hz (Supporting Figure S1). For practical flexible electronics application, flexible devices should be bendable without sacrificing their electrical properties. The influence of mechanical bending on the electrical characteristics of our devices was investigated. Figure 2b shows the transfer curves recorded before, during and after bending by a cylinder with a radius of 1.0 cm. The images of the measurement process are shown in the insets of Fig. 2b. Good reproducibility is obtained on different test conditions. Moreover, mechanical stress tests have also been performed by bending the sample repeatedly. Figure 2c shows the transfer curves recorded at repetitive bending cycles. The flexible neuromorphic transistors survive after more than 1000 flex/flat cycles with negligible change in the transfer characteristics. The variations in V_th_ and μ_FE_ with the repetitive bending cycles are extracted, as shown in Fig. 2d. After 1000 cycles of bending and recovery, a small positive shift of ~0.1 V in V_th_ and only ~10% reduction in μ_FE_ are measured. The results indicate that the flexible neuromorphic transistors have good mechanical reproducibility and durability.

**Figure 3:**
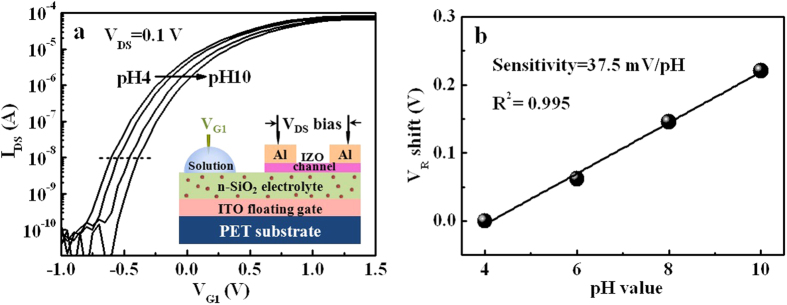
pH sensitivities of the IZO-based neuromorphic transistor measured in single-gate mode. (**a**) Transfer curves of the neuromorphic transistor measured by using the sensing gate G_1_ at V_DS_ = 0.1 V. The pH value of the solution droplet on the sensing gate G_1_ is changed from 4 to 10 at a step of 2. The inset shows the measurement schematic. (**b**) The sensitivity in terms of V_R_ shift. The data can be fitted linearly by the black line. A sensitivity of ~37.5 mV/pH and a linearity of ~0.995 are obtained.

We will next study the pH sensing performance of the devices operated in the quasi-static mode. Figure 3a shows the transfer curves of the neuromorphic transistor based sensor operated at the linear region (V_DS_ = 0.1 V) with the sensing gate immersed into solution droplets with different pH values. The inset in Fig. 3a shows the layout of this normal pH sensing measurement. Clear negative shift of the transfer curve is observed when pH value decreases from 10 to 4. It has been reported that acidic solution can give rise to a more positive surface potential due to the ionic interaction at the solution/SiO_2_ interface^38^,^39^. In our case, positive surface potential will make protons within SiO_2_ electrolyte migrate to the electrolyte/IZO channel interface, which will induce excess electrons in the IZO channel and a negative shift of transfer curve. When the gate voltage at a drain current of 10 nA is defined as the responsive voltages (V_R_), a sensitivity of ~37.4 mV/pH is realized, as shown in Fig. 3b. This value is comparable to the reported FET sensors using SiO_2_ as a sensing material^40^.

**Figure 4:**
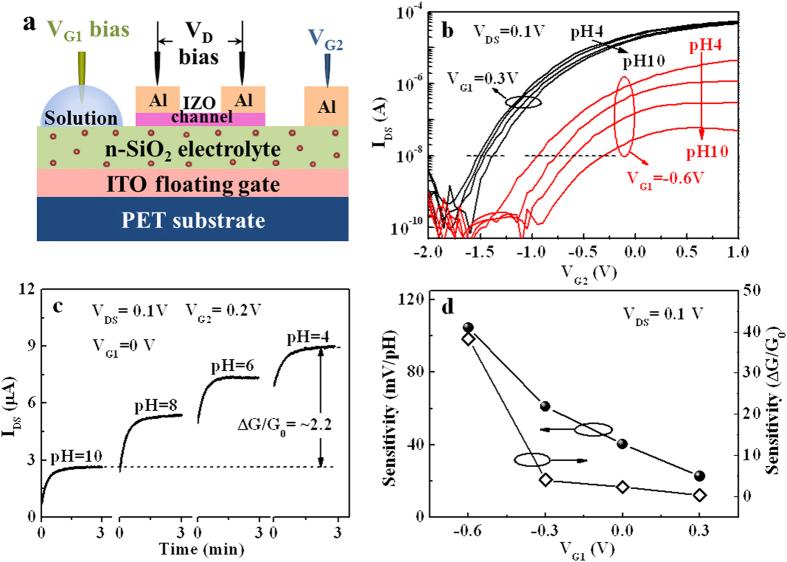
pH sensitivities of IZO-based neuromorphic transistor measured in dual-gate synergic modulation mode. (**a**) The schematic image of the measurements. (**b**) Transfer curves of the device measured by applying sweep voltage on the control gate (G_2_) at V_DS_ = 0.1 V with different fixed voltages applied on G_1_. pH value of the solution droplet on G_1_ increases from 4 to 10 at a step of 2. (**c**) The real-time responses of I_DS_ for IZO neuromorphic transistor sensors in each pH solution for 180s at V_DS_ = 0.1V, V_G1_ = 0V and V_G2_ = 0.2V. (**d**) The sensitivity in terms of V_R_ shift and ΔG/G_0_ at different V_G1_.

In order to improve the sensing performance of the IZO-based neuromorphic transistor, dual-gate synergic modulation mode is investigated. During the measurements, G_1_ is biased at different fixed voltages and G_2_ is swept from –2.0 to 1.0 V. The measuring schematic is shown in Fig. 4a. Figure 4b shows the transfer curves (I_DS_-V_G2_) curves measured at V_DS_ = 0.1 V with pH value changed from 10 to 4 and V_G1_ fixed at 0.3 V and −0.6 V, respectively. Similarly, the transfer curves shift to the negative direction when the pH value decreases at a fixed V_G1_. Here, we should point out that more obvious shifts in the transfer curve are induced by pH variation when V_G1_ = −0.6 V. The sensitivity in terms of V_R_ shift is plotted as a function of V_G1_ (Fig. 4d). The pH sensitivity increases when V_G1_ shifts from a positive value to a negative value. A maximal pH sensitivity of ~105 mV/pH is obtained when V_G1_ = −0.6 V. The improved sensitivity obtained at a negative V_G1_ is attributed to amplified capacitive coupling factor between these two gates (G_1_ and G_2_). Asymmetric dual-gate capacitive coupling can result in intrinsic amplification of the measured surface potential shifts. Theoretical analysis of the quasi-static pH sensing process can be found in Supporting Note 1. Jayant *et al.* also reported that this technique merely scaled the surface potential shift, but did not signify any change in the intrinsic properties of the electrolyte interface^21^. Figure 4c shows real-time responses of I_DS_ of the IZO-based neuromorphic transistor sensor in different pH solutions for 180 s at fixed V_DS_ = 0.1V, V_G1_ = 0 V and V_G2_ = 0.2V. It is observed that I_DS_ increases gradually to a stable value. The steady I_DS_ increases stepwise with discrete changes in pH value from 10 to 4. The sensitivity S of a sensor can also be defined as the relative change in channel conductance, S = (|G−G_0_|)/(G_0_) = ΔG/G_0_^41^. In our case, the response conductance to pH = 10 is defined as G_0_. Therefore, the sensitivity (∆G/G_0_) is estimated to be ~2.2 for pH = 4 at equilibrium state. We also find that the sensitivity (∆G/G_0_) can be improved by a negative bias applied on sensing gate (G_1_), as shown in the right axis of Fig. 4d. A highest sensitivity of ~38.3 is obtained at a fixed V_G1_ of −0.6 V. This value is much higher than those reported in nanoscale transistor sensors^42^,^43^. This is because that an appropriate negative voltage applied on the sensing gate (G1) can make the neuromorphic transistor operated in the subthreshold regime, in which the sensitivity in terms of current variation can be exponentially enhanced due to the most effective gating effect of charges bound on a surface^44^.

**Figure 5:**
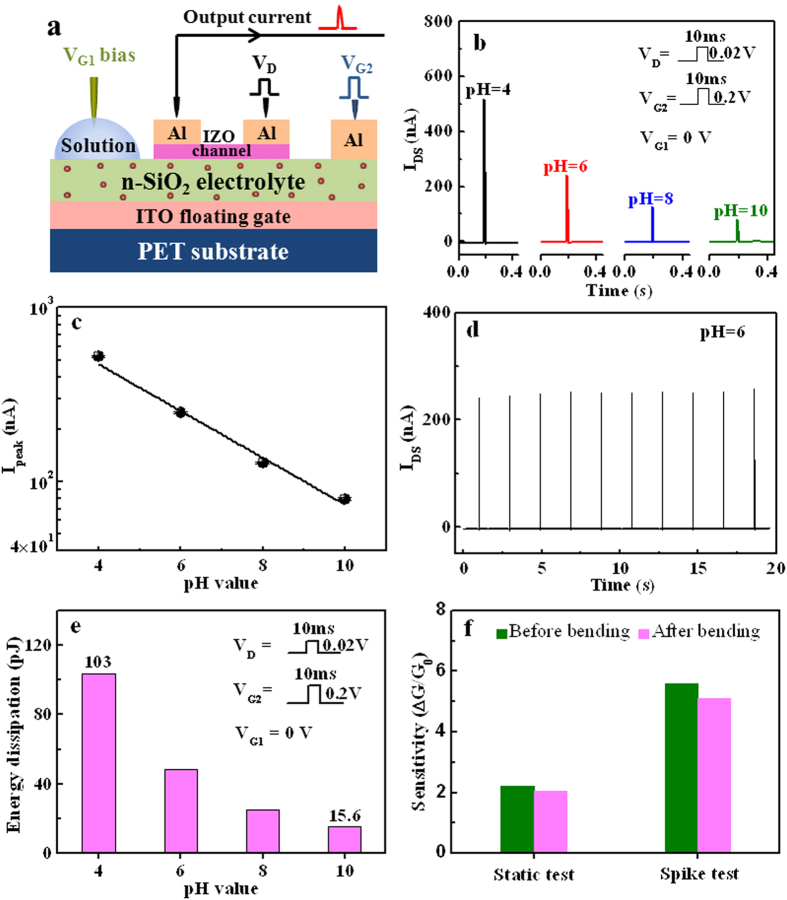
pH sensing performances of IZO-based neuromorphic transistor operated in a single-spike mode. (**a**) Schematic diagram of single-spike pH sensing measurements. (**b**) Single-spike measurement is performed for pH value increases from 4 to 10. The spike voltage V_G2_ (0.02V, 10 ms) and the reading voltage V_D_ (0.2 V, 10 ms) are applied synchronously. The reference electrode V_G1_ is grounded. (**c**) The logarithm of I_DS_ peak changes linearly with the pH value of the solution. The error bars represent standard deviations for 10 samples. (**d**) Reproducibility of the neuromorphic transistor sensor with pH = 6. (**e**) pH value dependent energy dissipation operated in single-spike mode. (**f**) The influence of 1000 times bending on the sensitivity of the neuromorphic transistor for both quasi-static and single-spike sensing modes. Fixed biases (V_DS_ = 0.1 V, V_G1_ = 0 V, V_G2_ = 0.2 V) are applied in quasi-static mode. Synchronous pulse voltages V_G2_ (0.2 V, 10 ms) and V_D_ (0.02 V, 10 ms) with fixed V_G1_ bias of 0 V are applied in dynamic spiking mode.

Next, inspired by the spiking operation mode of a biological neuron, we have investigated the single-spike pH sensing performance of our neuromorphic transistor sensors. Due to the distinctive dynamic characteristics of the proton migration, our device presents a unique time dependent transient property. During the measurement, equilibrium is disturbed by a small voltage pulse applied on the control gate (G_2_). The dynamic spike current response to such a disturbation contains the pH sensing information. After the detection, the device will quickly recover to the original equilibrium state. Moreover, during the single-spike sensing process, the energy consumption is extremely low, which is preferred for portable and wearable sensory applications. The single-spike pH sensing measurement of the detection is schematically illustrated in Fig. 5a. At first, a disturbing spike V_G2_ (0.2 V, 10 ms) was applied on control gate (G_2_), and a synchronous reading spike V_D_ (0.02 V, 10 ms) was applied on drain electrode to measure the output current. As shown in Fig. 5b, when the pH value is changed from 4 to 10, the response current (I_DS_) decreases from 512 to 80 nA. We also find that the logarithm of I_DS_ decreases linearly with increasing pH value, and a high sensitivity (∆G/G_0_) of ~5.6 is estimated, as shown in Fig. 5c. The characteristic time of the dynamic process of proton migration in the nanogranular SiO_2_ electrolyte is in the order of few milliseconds. The response/recover time is estimated to be ~5.0 milliseconds, which is much shorter than that operated in quasi-static mode. The reproducibility of the single-spike pH sensing measurement is also investigated. Figure 5d shows the response currents stimulated by repeated voltage pulse spikes with pH = 6. The results indicate a good reproducibility of single-spike detection of pH values. If we define the value of σ(I_p_)/Ave(I_p_) as the noise factor, where σ(I_p_) is the standard variation of the repeated spike current peaks, and Ave(I_p_) is the average value of repeated spike current peaks. The value of σ(I_p_)/Ave(I_p_) is calculated to be only ~1.7% for pH = 6. Detailed analysis of the reproducibility and noise of the spike sensing can be found in Supporting Note 2. The power consumption of our system can be estimated by multiplying the reading voltage, the channel current and the spike duration time^45^. Figure 5e shows the average energy dissipation for single-spike pH detection in each pH value from 10 to 4 with a spike duration time of 10 ms. The power consumption reduces from 103 pJ/spike to 15.6 pJ/spike when the pH value increases from 4 to 10. Of course, the power consumption can be further reduced by reducing the spike voltage and spike duration time. The influence of bending on the sensitivity is also investigated. As shown in Fig. 5f, after 1000 bending cycles, the sensitivity reduction is less than 10% for both quasi-static and single-spike sensing modes.

**Figure 6:**
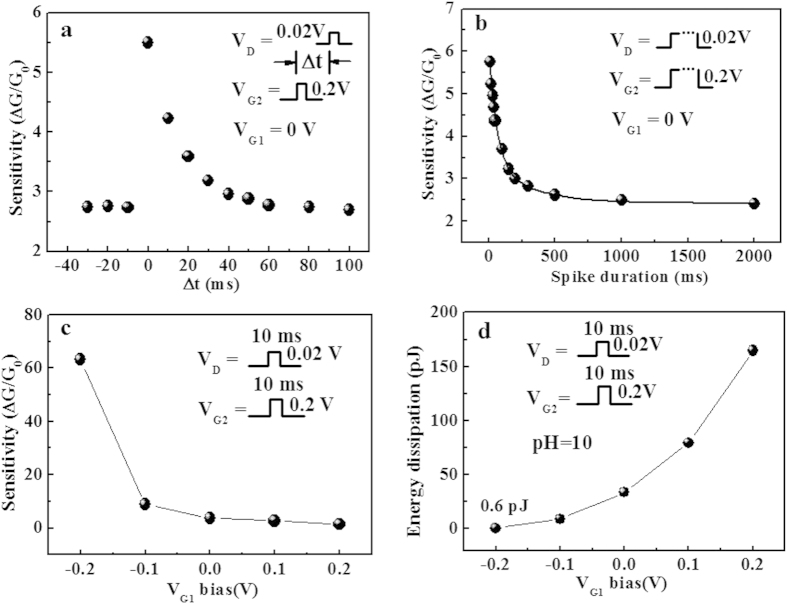
Influences of measuring parameters on the spike sensing performance. (**a**) The sensitivity as a function of the inter-spike interval between V_D_ and V_G2_ for asynchronous spiking sensing test. (**b**) The changes in sensitivity with spike duration. The solid line is the fitted curve. (**c**) The changes in sensitivity and energy dissipation (pH = 10) with various V_G2_ spike amplitude for synchronous spiking sensing test. (**d**) The changes in sensitivity and energy dissipation (pH = 10) against various V_G1_ bias.

The single-spike sensing performance implemented with an asynchronous reading spike is also investigated. Figure 6a shows the sensitivity as a function of the inter-spike interval (Δt) between V_D_ and V_G2_. If Δt < 0, the reading spike V_D_ is applied before V_G2_. In this case, the protonic disturbance does not happen in the sensing process, thus the sensitivities are close to the equilibrium state and a sensitivity (∆G/G_0_) of ~2.7 is obtained. When Δt ≥ 0, the measured sensitivity is time interval (Δt) dependent. A highest pH sensitivity (∆G/G_0_) of ~5.6 is obtained when Δt = 0 and it gradually reduces to 2.7 with increasing Δt. Figure 6b shows the sensitivity as a function of spike duration time. At present, in order to accurately measure a low current in the nA scale by semiconductor analyzer, the shortest spike duration we can used is 10 ms. In this case, the maximal pH sensitivity (∆G/G_0_) is measured to be ~5.6. We can anticipate that the sensitivity can be improved further when the spike duration time is reduced further. Detailed theoretical analysis of the influence of spike duration on the sensitivity can be found in Supporting Note 3. We also find that the sensitivity decreases gradually to ~2.4 when the spike duration increases to 2 s. These results indicate that the neuromorphic transistor sensor tends to arrive at equilibrium state with the increase of spike duration. The sensitivity of single-spike pH sensing performances of our neuromorphic transistor sensor can be improved further by additional gate synergic modulation. Figure 6c shows the influence of voltage bias applied on G_1_ on the sensitivity when the device is operated in single-spike mode. The pH sensitivity increases when V_G1_ shifts from positive to negative. A maximum sensitivity (∆G/G_0_) of ~63 can be obtained when a negative voltage of −0.2 V is applied on G_1_. We also investigated the influence of voltage bias applied on V_G1_ on the energy dissipation of single-spike sensing measurement. Our results indicate that the energy dissipation can be gradually reduced when the V_G1_ is changed from 0.2V to −0.2 V. As shown in Fig. 6d, an ultra-low energy dissipation of ~0.6 pJ/spike is estimated for pH = 10 at V_G1_ = −0.2 V with the spike duration of 10 ms. Similar to the quasi-static synergic mode, an appropriate negative V_G1_ can make the device operate in the subthreshold regime. Thus, an enhanced sensitivity can be obtained. At the same time, negative bias can reduce the spike sensing current, which is critical for energy dissipation reduction.

The use of EDL electrolyte as gate dielectrics in flexible neuromorphic transistors can obviously reduce the operation voltage down to 2.0 V. Our results also demonstrate that spiking operation could greatly reduce the power consumption because the device is usually biased at zero voltage and only low voltage spikes with very short duration time are applied. Such neuromorphic transistors are favorable for flexible and portable sensor applications. Inspired by biological neuron, our neuromorphic transistor is designed with multiple in-plane gates. At present, we only investigate the influence of the second gate on the sensing performances in both quasi-static and spiking modes. Such devices can also be proposed as multi-functional sensors, where one in-plane gate acts as modulation terminal, one in-plane gate acts as calibration terminal, and other in-plane gates act as sensing input terminals. In the future, multiple-gate stochastic resonance effects may also be explored for further sensitivity improvements and power consumption reductions when such multiple-gate devices are operated in neuromorphic sensing mode.

In summary, flexible oxide-based neuromorphic transistors were fabricated on plastic substrates. A pH sensitivity of ~105 mV/pH was obtained for quasi-static dual-gate synergic sensing mode. Our results demonstrated that single-spike dynamic sensing mode could remarkably improve the pH sensitivity and reduce response/recover time and power consumption. We also found that appropriate depression voltage applied on the sensing gate could further enhance the pH sensitivity and reduce the power consumption. Our results provided a novel strategy for fabricating biochemical sensors with high sensitivity, rapid response and ultralow power consumption.

## Methods

### Fabrication of flexible oxide-based neuromorphic transistors

First, 500-nm-thick nanogranular SiO_2_ electrolyte films were deposited on ITO-coated PET substrates by plasma enhanced chemical vapor deposition (PECVD) at room temperature. SiH_4_ (95% SiH_4_ + 5% PH_3_) and pure O_2_ were used as reactive gases. Then, 30-nm-thick IZO channel layer was sputtered on the SiO_2_ electrolyte films by using a nickel shadow mask. The sputtering was performed at a RF power of 100 W and a working pressure of 0.5 Pa using an IZO target. The channel width and length were 1000 and 80 μm, respectively. Finally, 100-nm-thick Al source/drain electrodes and in-plane gate electrodes were deposited by thermal evaporation patterned by another shadow mask.

### Preparation of pH solution

pH solutions were prepared by titrating 10 mM phosphate solution with dilute hydrochloric acid or potassium hydroxide solutions. The pH value of the solutions was monitored by a commercial pH meter. All chemicals were purchased from Sinopharm Chemical Reagent Co., Ltd. (China). Such phosphate buffered solutions were used for measurements due to their strong buffer capacity to the influence of external environment. Thus, the changes in pH signal due to relaxation of charges in oxide can be ignored.

### Electrical and sensing performance characterizations

The sensing area of the device was immersed in deionized water for 24 hours before measurements. Frequency-dependent capacitances of the SiO_2_ electrolyte films were characterized by a Solartron 1260A Impedance Analyzer in air ambient with a relative humidity of ~55%. Transistor characteristics and pH sensing performance were recorded by a semiconductor parameter characterization system (Keithley 4200 SCS) at room temperature. After each pH value test, the solution droplet was removed and the sensing area was rinsed two times in deionized water.

## Additional Information

**How to cite this article**: Liu, N. *et al.* Flexible Sensory Platform Based on Oxide-based Neuromorphic Transistors. *Sci. Rep.* **5**, 18082; doi: 10.1038/srep18082 (2015).

## Supplementary Material

Supplementary Information
