# Supplementary material for: Flexible Sensory Platform Based on Oxide-based Neuromorphic Transistors
Source: Sci Rep. 2015 Dec 11;5:18082. doi: 10.1038/srep18082 (PMC4676022; doi:10.1038/srep18082)
Supplement: Supplementary Information [file srep18082-s1.doc]

**Supporting Information**

**Flexible Sensory Platform Based on Oxide-based Neuromorphic Transistors**

Ning Liu^1, 2)^, Li Qiang Zhu^2)^, Ping Feng^1)^, Chang Jin Wan^2)^, Yang Hui Liu^2)^, Yi Shi^1)^ and Qing Wan^1)^

^1)^ School of Electronic Science & Engineering, Nanjing University, Nanjing 210093, People’s Republic of China.

^2)^ Ningbo Institute of Materials Technology and Engineering, Chinese Academy of Sciences, Ningbo 315201, People’s Republic of China.

To whom correspondence can be addressed, E-mail: [wanqing@nimte.ac.cn](mailto:wanqing@nimte.ac.cn) [yshi@nju.edu.cn](mailto:yshi@nju.edu.cn)


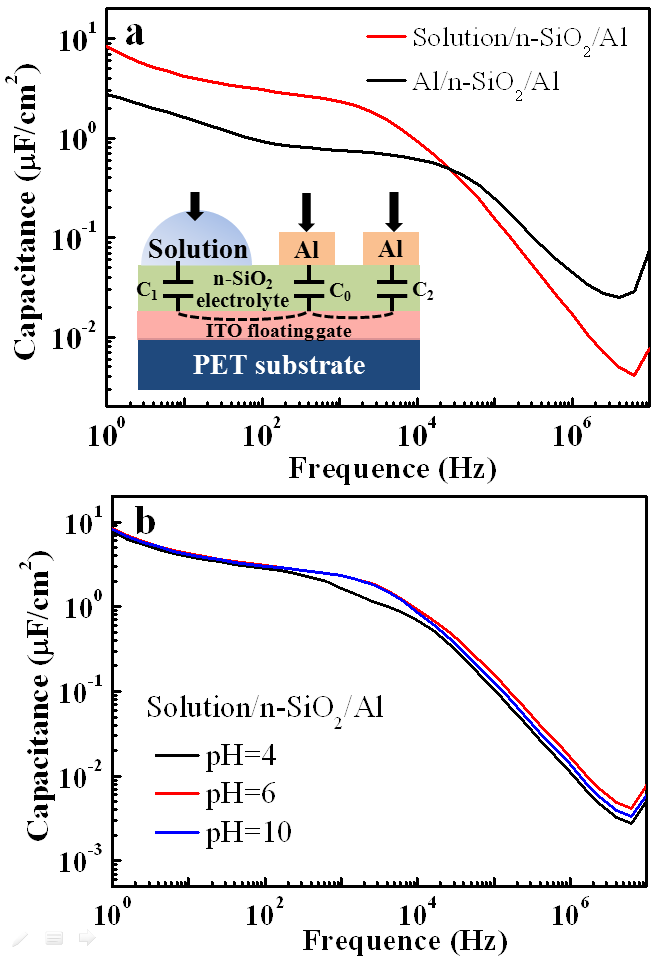


**Supporting Figure S1 |** **Capacitance-frequency curves of the SiO_2_ electrolyte films.** **(a)** The specific capacitance–frequency curves for the SiO_2_ electrolyte films on PET substrate with a liquid droplet (pH=6) and an Al electrode, using an in-plane test structure. Inset shows the schematic diagram of the capacitance measurement. The specific capacitances at 1.0 Hz are estimated to be ~8.3 μF/cm^2^ and ~2.7 μF/cm^2^ for liquid droplet and Al electrode, respectively. **(b)** The specific capacitance–frequency curves for the SiO_2_ electrolyte films gated by sensing gate with different pH solutions (pH= 4, 6, 10). Little difference among the C-f curves at different pH solutions is observed.

**Supporting Note 1. Theoretical analysis of quasi-static pH sensing process**

According to the celebrated site-binding theory and Gouy-Chapman-Stern model [45, 46], the V_th_ shift of FET-based sensor results from a change in surface potential (*φ*_0_) at the interface between the solution and ion sensing membrane. The relation between the surface potential (*φ*_0_) and the pH value of the solution is given by:

(1)

Where *k* is the Boltzmann constant, T is the absolute temperature, *q* is the elementary charge, and *α* is a dimensionless sensitivity parameter which varies between 0 and 1. When the sensitivity parameter α approaches to unity, the sensing membrane shows a maximum pH sensitivity of 59.2 mV/pH at room temperature (298 K), which is known as the Nernst limit.

When the IZO-based neuromorphic transistor sensor is operated in quasi-static dual-gate mode, the effective capacitance network of is proposed in **Figure S2**, where C_GCDL_ is defined as the capacitance for Helmholtz double layer and Gouy–Chapman diffuse double layer of the solution in series. C_0_, C_1_, C_2_ are the EDL capacitances of SiO_2_ electrolyte at the interface of IZO channel, G_1_, and G_2_, respectively. It is observed that G_1_ and G_2_ are capacitively coupled to the bottom ITO conductive layer, which is analogous to the floating gate (FG) of neuron MOS (νMOS) [15]. Therefore, the ITO FG voltage (V_FG_) can be defined by the weighted sum of the gate inputs, and given by [47]:

(2)

Here, C_tot_ is the total capacitance seen by the floating gate. It includes all other parasitic capacitances that are ignored here. It is customary to assume that the FG has no initial charges.

Referring to equation (1) and (2)，the sensitivity of V_FG_ to pH values can be deduced as:

$\frac{dV_{FG}}{dpH}=\frac{C_{1}}{C_{tot}}\times2.3\alpha\frac{kT}{q}$ (3)

When G_2_ is used as the control gate with a constant V_G1_, then the pH sensitivity referred to V_G2_ is given by:

$\frac{dV_{FG}}{dV_{G2}}=\frac{C_{2}}{C_{tot}}$ (4)

(5)

where Δ*ϕ*_0_ is the change in surface potential induced by pH variations. *η* is the factor representing the coupling ratio between these two gates. Thus, the measured surface potential shift is scaled by asymmetric capacitive coupling between G_1_ and G_2_. In quasi-static measurement, when a negative bias is applied to G_1_, more protons in the SiO_2_ electrolyte will accumulate close to G_1_. Thus, C_2_ will reduce due to the less amounts of protons regulated by G_2_. Therefore, an amplified capacitive coupling ratio (C_1_/C_2_) is obtained with a negative V_G1_, achieving a higher pH sensitivity. In contrast, a positive V_G1_ gives rise to an increase of C_2_, and then leads to a lower pH sensitivity.


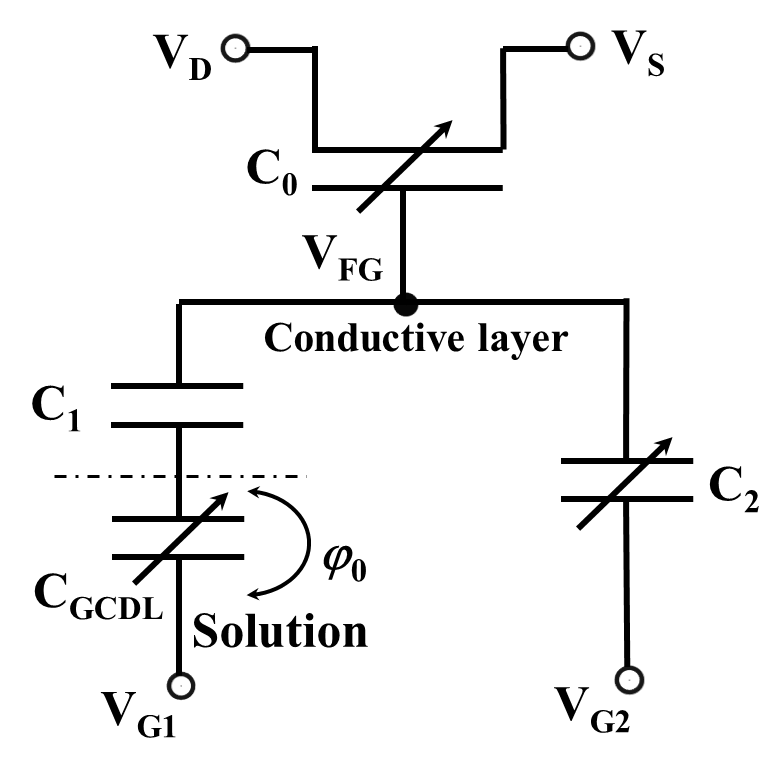


**Supporting Figure S2 | The effective capacitance network of the flexible IZO-based transistor sensors.**

For constant voltage mode (I_DS_-time measurements), the current is exponential to the gate voltage in the subthreshold region,

$I_{DS}\sim\exp[\frac{q(V_{GS}-V_{th})}{kT}]$ (6)

Referring to equation (5) and (6), the sensitivity of dual-gate transistor sensors in terms of ΔG/G_0_ at equilibrium state can be deduced as:

(7)

Where G_a_ and G_b_ are the channel conductance of the neuromorphic transistor sensors measured with acidic and alkaline solution, respectively. It can be seen that the sensitivity will be exponentially enhanced with the decrease of pH values in the substhreshold region.

**Supporting Note 2. Reproducibility and noise analysis of spike sensing**

In the case of spike sensing mode, several spiking output currents were recorded, and it exhibit good reproducibility for pH sensing at different pH solution, as shown in **Figure S3**. We define the value of σ(I_p_)/Ave(I_p_) as the noise factor, where σ(I_p_) is the standard variation of the repeated spike current peaks, and Ave(I_p_) is the average value of repeated spike current peaks. The value of σ(I_p_)/Ave(I_p_) is calculated to be only ~1.2%, 1.7%, 1.2%, 1.6% for pH=4, 6, 8, 10, respectively. The results indicate that spiking sensing mode is of great importance to improve the sensitivity and SNR.

**Figure S3 |** The synaptic output spike current is recorded versus time to exhibit the reproducibility of the spike pH sensing measurement for different pH value of the solution: (a**)** pH=4;(b**)** pH=6;(c) pH=8;(d) pH=10. Pre-synaptic spikes V_G1_ (0.2 V, 10 ms) and synchronous reading spikes V_D_ (0.02 V, 10 ms) were applied on the dendritic input and output, respectively.

**Supporting Note 3. Theoretical analysis of single-spike pH sensing process**

In the spike sensing mode, the channel current is induced by the ionic diffusion due to the EDL effect. The EDL capacitor can be modeled as an ideal resistor-capacitor (RC) circuit. The effective voltage on the EDL capacitor is given by:

$V_{E}=V_{GS}[1-\exp(-\frac{t}{\tau})]$ (8)

where t is the voltage bias duration, *τ* is the time constant of equivalent RC circuit of the EDL capacitor. According to (6) and (8), the subthreshold current is

$I_{DS}\sim\exp\{\frac{qV_{GS}(1-\exp(-t/\tau)-qV_{th}]}{kT}\}$ (9)

Thus, the sensitivity in terms of ΔG/G_0_ for the spiking mode is

(10)

where *τ*_a_ and *τ*_b_ are the time constant of the EDL capacitor for the neuron transistor sensor under acidic and alkaline solution, respectively. Defining a proportion function $f(t,\tau)=\exp(\frac{qV_{GS}}{kT}[\exp(-\frac{t}{\tau_{b}})-\exp(-\frac{t}{\tau_{a}})])$, the sensitivity in terms of ΔG/G_0_ for spiking mode can be approximated to be:

(11)

For a short spike duration, if*τ*_a_<*τ*_b_, *f*(t, *τ*) >1, the sensitivity is enhanced. If *τ*_a_>*τ*_b_, *f*(t, *τ*) <1, the sensitivity is reduced. For a long spike duration, *f*(t, *τ*) →1, it is equal to the sensing process at equilibrium state.

The value of *τ* reflects the transporting speed of protons in n-SiO_2_ electrolyte films. At a lower pH value, acidic solution gives rise to a more positive surface potential, which promotes the transporting of protons within n-SiO_2_ electrolyte films, and leads to a smaller *τ* value. Hence, an amplified factor (*f*(t, *τ*)>1) for pH sensitivity can be realized in the spike sensing mode. The *τ* values can be deduced by fitting the transient current of IZO neuron transistor sensors as a function of time, as shown in the Fig. S4.

**Supplementary Figure S4 |** **The transient current versus time for different pH values. (a)** pH=4; **(b)** pH=6; **(c)** pH=8; **(d)** pH=10. Fixed voltage biases (V_DS_=0.2V, V_G1_=0V, V_G2_=0.2V) are applied. The transient currents can be well fitted by equation (6), as shown in the red solid lines. The fitted *τ* is estimated to be 0.26s, 0.28s, 0.30s and 0.33s for pH value of 4, 6, 8 and 10, respectively.

**Supplementary References**

1. Yates D. E. L., S.; Healy, T. W. Site-binding model of the electrical double layer at the oxide-water interface. *J. Chem. Soc. Faraday Trans.* **70**, 1807-1818 (1974).
2. Bousse, L., De Rooij, N. F. & Bergveld, P. Operation of chemically sensitive field-effect sensors as a function of the insulator-electrolyte interface. *IEEE Trans. Electron Devices* **ED-30**, 1263-1270 (1983).
3. Al-Ahdal, A., Toumazou, C. High gain ISFET based νMOS chemical inverter. *Sens. Actuators B* **171**, 110-117 (2012).
